# Nano-particle mediated inhibition of Parkinson’s disease using computational biology approach

**DOI:** 10.1038/s41598-018-27580-1

**Published:** 2018-06-15

**Authors:** Aman Chandra Kaushik, Shiv Bharadwaj, Sanjay Kumar, Dong-Qing Wei

**Affiliations:** 1 State Key Laboratory of Microbial Metabolism and School of life Sciences and Biotechnology, Shanghai Jiao Tong University, Shanghai, 200240 China; 2 Sabanci University Nanotechnology Research and Application Center, Orta Mah. Tuzla, 34956 Istanbul, Turkey; 3 Bioinformatics Centre, Biotech Park, Lucknow, 226018 India

## Abstract

Parkinson’s disease (PD) arises as neurodegenerative disorder and characterized by progressive deterioration of motor functions due to forfeiture of dopamine-releasing neurons. During PD, neurons at stake loss their functionality that results into cognition impairment and forgetfulness, commonly called as dementia. Recently, nanoparticles (NPs) have been reported for easy drug delivery through blood-brain barrier (BBB) into the central nervous system (CNS) against the conventional drug delivery systems. However, present study attempted to elucidate the α-synuclein activity, a major factor casing PD, in presence of its inhibitor cerium oxide (CeO_2_) nanoparticle via computational biology approach. A computational analysis was also conducted for the α-synuclein activity with biocompatible metal NPs such as GOLD NPs and SPIONs to scrutinize the efficacy and degree of inhibition induced by the CeO_2_ NP. The obtained results concluded that CeO_2_ NP fit best in the active site of α-synuclein with good contacts and interaction, and potentially inhibited the PD against L-DOPA drug selected as positive control in the designed PD biochemical pathway. Hence, CeO_2_ NP has been purposed as potential inhibitor of α-synuclein and can be employed as nano-drug against the PD.

## Introduction

Neurodegenerative diseases (NDs) such as Alzheimer’s disease (AD), Multiple sclerosis (MS), Parkinson’s disease (PD) and Amyotrophic lateral sclerosis (ALS) following viral infections, are multifaceted disorders occurred due to amalgamation of both genetic and environmental factors on set of aging^1,2^. PD is most commonly and progressive ND recorded with increasing prevalence in elderly population^3^, PD have been reported to effect approximately 3% population over the age of 65, and stand second only to Alzheimer’s disease^4,5^. Clinical diagnosis of PD is generally characterized by selective neurodegeneration or death of mesencephalic dopaminergic (mDA) neurons in the substantia nigra pars compacta (SNpc) that results into subsequent reduction of dopamine in striatum as significant neuropathological hallmark^3,6^. Moreover, incidence of intracellular diseases-related protein aggregates, known as Lewy bodies and Lewy neurites also defined the neuropathological feature of PD present in persisting dopaminergic neurons of SNpc. The filaments of Lewy bodies and Lewy neurites were reported to contain α-synuclein as major component^7^. Also, missense mutations (such as A53T, A30P, and E46K) in the α-synuclein gene (*PARK1*) along with duplications as well as triplications of α-synuclein gene (initially *PARK4*) containing locus was reported to cause rare familial forms of PD^6^. Additionally, gene polymorphisms have been classified as risk factors for idiopathic or sporadic PD^8,9^.

In present scenario, efforts have been made for the development of pharmacological approaches against PD such as drugs that can accelerate intracerebral dopamine levels and/or stimulates central dopamine receptors. For instance, dopamine precursor substance L-3,4-dihydroxyphenylalanine (L-DOPA) has been approved as an optimal dopamine substitution therapy in combination with peripheral dopamine decarboxylase inhibitor (Benserazide or Carbidopa)^10^. However, these drugs are limited to DAergic targeting therapeutic strategy and solely ameliorated the symptoms whilst there is little or no evidence documented that such therapies assisted in retardation of DA neuron degeneration^11^. Additionally, drug therapies such as L-DOPA was reported to induced several adverse side effects which includes arrhythmia, gastrointestinal discomfort, extreme emotional variability with prevalent anxiety, hallucinations, impaired social behaviour, excessive libido and compulsive behaviour^12^. Moreover, neuroprotection trials till date have not yet clearly identified a drug that can delay or halt disease progression^13^. This lack in potential therapeutic against PD may be expectable because of several challenges includes; (i) early diagnosis of PD is impeded due to absence of efficient bio-markers, (ii) persistent neurodegeneration during PD often results into secondary effects such as chronic inflammation and (iii) possible resistance to drugs administered into the central nervous system against PD in the brain because of blood-brain barrier (BBB), and to target specific cell types with in different CNS regions demand an efficient vectors that can carry the therapeutic agents at the target sites as summarized earlier^14^. Under these circumstances, there is highly demand for the cheap and effective treatment which is more reliable with no or minimum side effects^15,16^.

**Figure 1:**
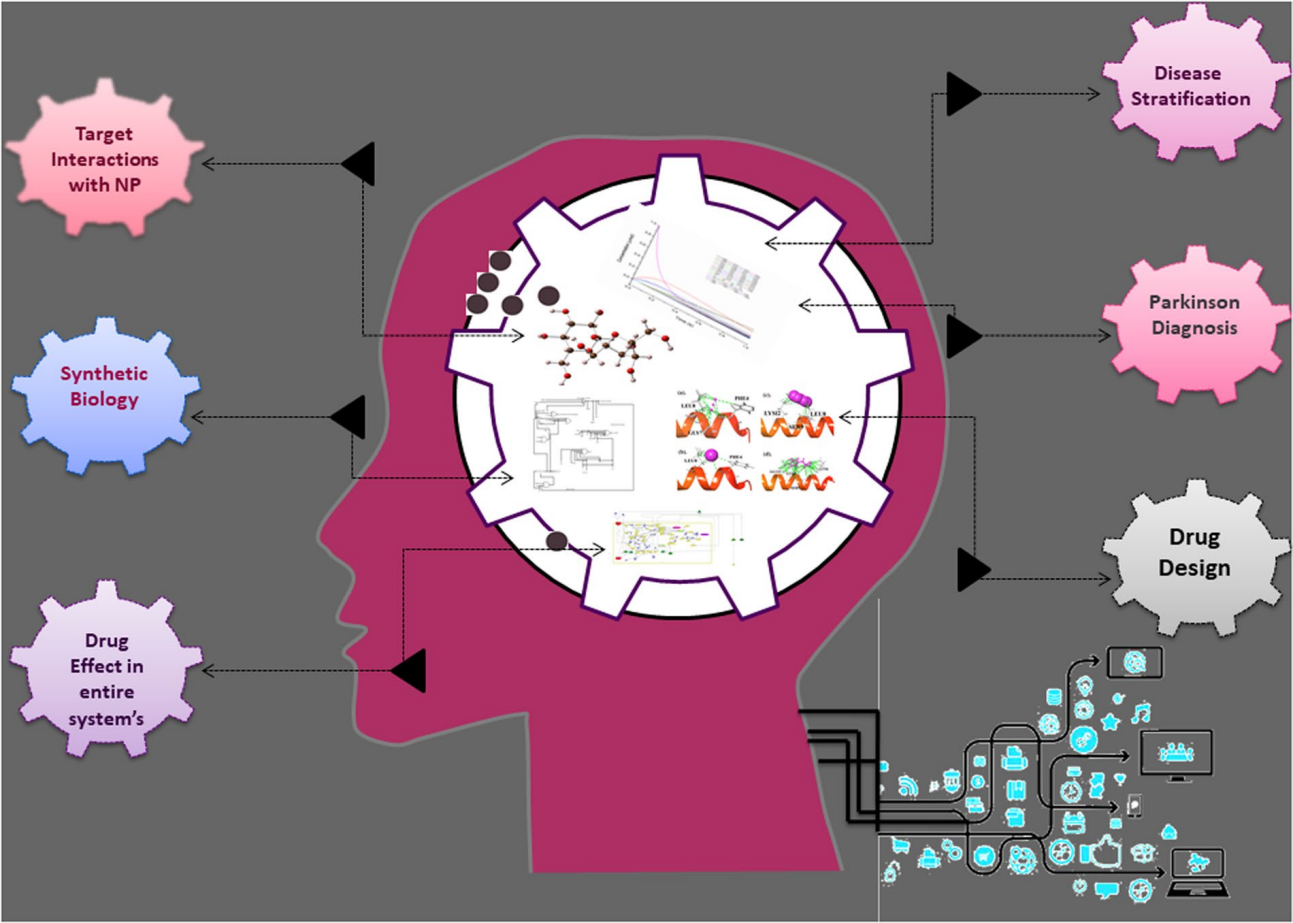
Schematic representation of different steps followed for nanoparticle-based drug development against PD using computational biology approach.

In this context, application of nanoparticles (NPs) has been emerged as revolutionary treatment against conventional drug delivery systems for neurodegenerative diseases such as PD due to their site directed target delivery and ability to pass through BBB into central nervous system^17^. For instance, NPs surface functionalised with peptidomimetic antibodies has been reported as molecular Trojan horses to transport bulky molecules such as drugs and genes across the BBB^18,19^. However, biocompatible gold nanoparticles (AuNPs) has been described to induce a strong α-synuclein aggregation at 20 nM concentration^20^. Among other NPs, graphene^21,22^ and superparamagnetic iron-oxide nanoparticles (SPIONs) were also documented to inhibit the Aβ fibrillation process during NDs^23–25^. Whilst, in particular, cerium oxide (CeO_2_) NPs has been reported to show neuroprotective activity because of their antioxidant and anti-apoptotic effects^26,27^. Recently, biological network representations and biochemical mathematical models have been used to explain interactions (such as activation or inhibition of biological molecule activities and pathways between entities) and to revealed the pharmacokinetics mechanism in a specific systems^28^, respectively. The biological interactions are generally deigned based on literature survey for the selected entities under different conditions and properties^29,30^. Since, no such biochemical pathway has been documented till now for the PD pathogenesis, hence, designing of PD biochemical pathway and targeting of key molecules or genes involved in the pathogenesis may allow for the rational and disease-modifying drugs development against PD. Moreover, synthetic biology comprehends a whole new era of engineering approaches and has been incorporated as synthetic bio-models for curing diseases. For instance, biomedical theranostics and cancer treatment has been documented as rationalise method to encode the biological processes in the living system^31^. Such type of synthetic models can also be designed for PD and may be proved helpful in elucidating the gene regulation, protein forming machinery with respect to cascading effect and other biological interactions during PD pathogenesis. Hence, this study proposed a complete biochemical pathway for PD with respect to α-synuclein based on the available information regarding genes/proteins involved in the PD etiology. Moreover, we testified in-silico biophysical and biochemical interactions of well-known biocompatible NPs for α-synuclein inhibition against standard drug molecules. Further, a synthetic biological model was also purposed based on the predicted biological interactions from the biochemical pathway using computational approach. An outline for the different steps followed for the present work are shown in Fig. 1.

## Results and Discussion

### Molecular interaction of Nanoparticles with α-synuclein

To elucidate the activity of NPs as nano-drug on the genetically linked gene encoding most susceptible protein α-synuclein, we performed α-synuclein docking with selected NPs (CeO_2_, AuNP, SPION NP) and L-DOPA drug molecules as standard control (Table S1). We employed shape based complimentary algorithm for the NPs to conduct docking with α-synuclein as the selected NPs did not possess valid atoms types for energy and score-based dock studies. Following, only one conformation was selected from 20 conformations per molecules to analyze the best fitting conformations at the active site of α-synuclein.

**Figure 2:**
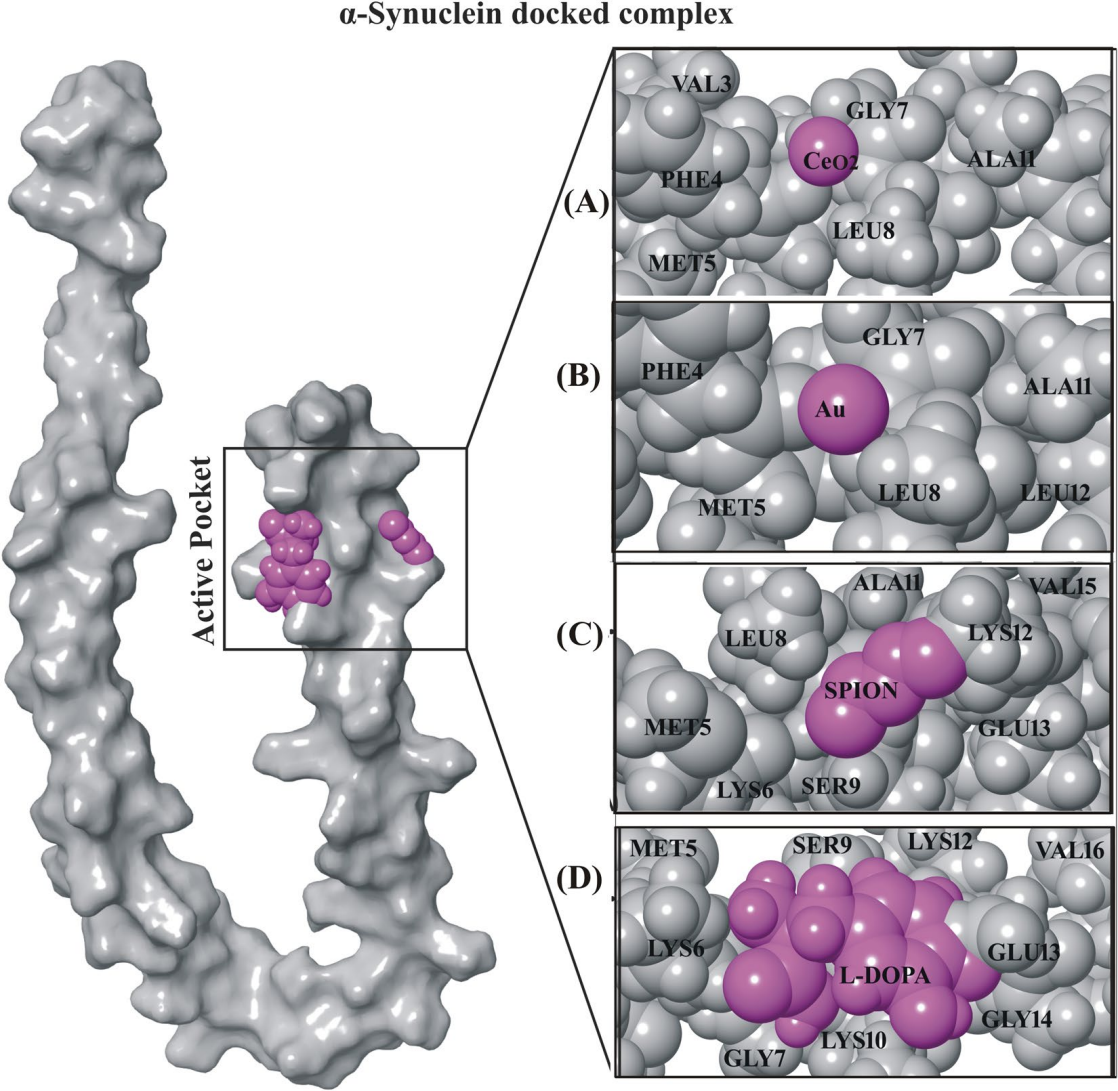
α-Synuclein docked complex with NPs and drug molecule. (**A**) CeO_2_NP (−2.1 kcal/mol)_,_ (**B**) Au NP (−0.4 kcal/mole), (**C**) SPION (−2.8 kcal/mol) and (**D**) L-DOPA (−3.5 kcal/mol) drug molecules. (NPs and drug molecule in magenta color with CPK representation and protein in light gray (25%) with CPK representation).

**Figure 3:**
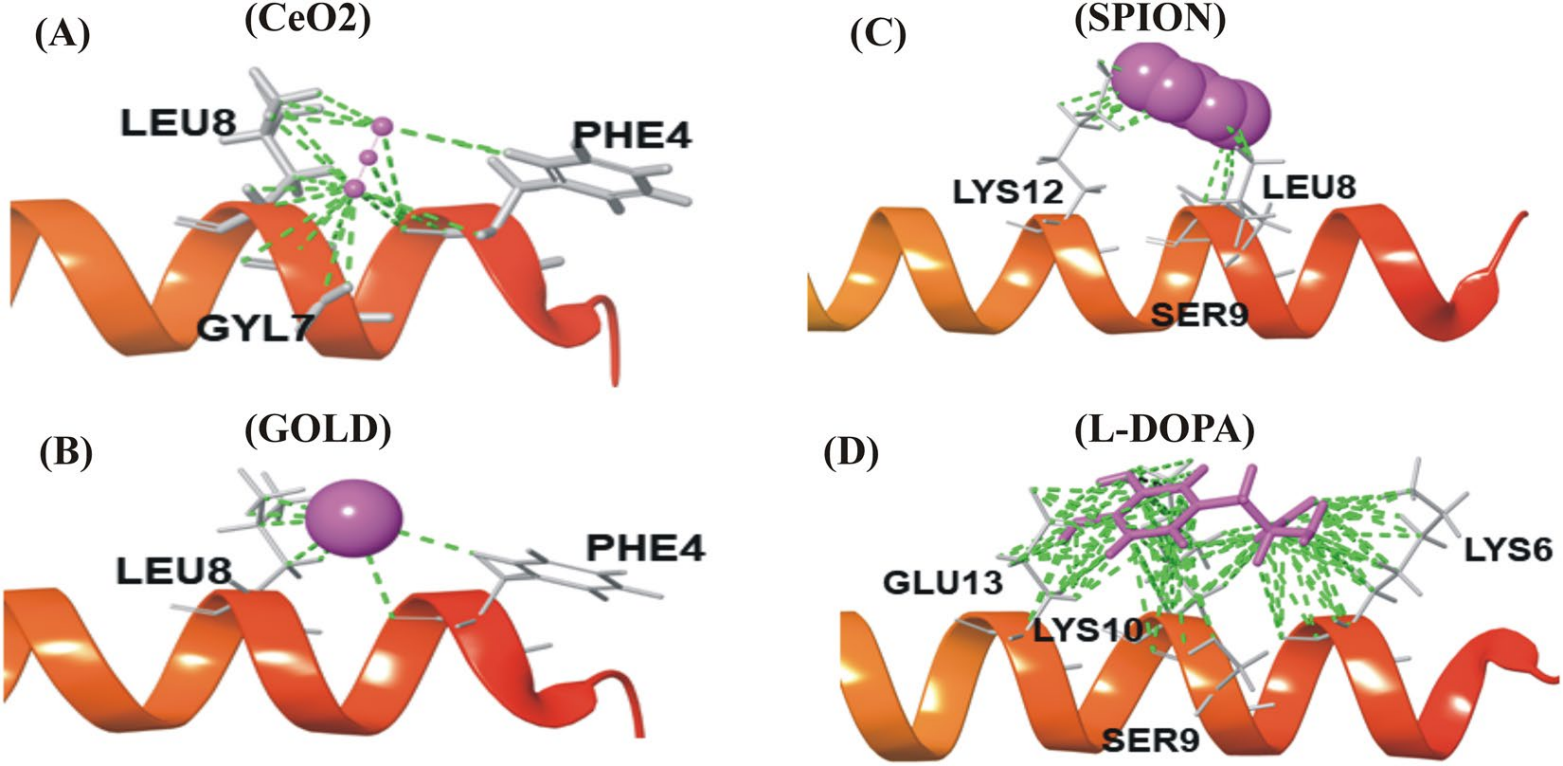
Nano-drugs along with standard control (**A**) CeO_2_ NP (**B**) AuNP, (**C**) SPION and (**D**) L-DOPA drug molecule depicting the good contacts (green color) at interacted regions (tube representation) with the α-synuclein protein (helix structure).

The docking results revealed that only CeO_2_ NP showed the best fitting in active site of α-synuclein (Fig. 2A). It was observed that cerium (Ce) and oxygen (O) atoms from CeO_2_ NP exhibited excellent metal interactions with the PHE4 and GLY7 residue, respectively of α-synuclein (Fig. 3A). Besides, both oxygen atoms from CeO_2_ NP showed good affinity towards PHE4, GLY7 and LEU8 residues of α-synuclein at a minimal distance of 4 Å (Fig. 3A). However, results from AuNP docking with α-synuclein did not show any interaction with PHE4 residue and LEU8 residue of α-synuclein at 4 Å distances (Figs 2B and 3B). Moreover, docking studies of SPION with α-synuclein displayed H-bonding only with LYE12 residue by oxygen atoms (O1 and O2) and iron (Fe) atoms along with all the oxygen atoms (O1, O2 and O3) in SPION depicted good contact with LEU8, SER9 and LYS12 residues (Figs 2C and 3C). Henceforth, these docking results nullifies the direct participation of AuNP and SPION in molecular interaction but respective oxygen atoms in their vicinity presented interactions with amino acid residues of α-synuclein. Moreover, it was also predicted that the Ce and Fe atoms inhibits the catalytic function of α-synuclein by forming a good metallic contact with its catalytic residues. Additionally, interaction of oxide atoms with hydrocarbon (HZ1 and HZ2) was also recorded to contribute in the stabilization of protein-metal interactions. Whilst, analysis of L-DOPA drug molecule, as standard control against nano-drugs, docked with α-synuclein protein showed H-bond interaction and hydroxyl group (-OH) donor-acceptor bond with LYS10 residue and amino group (NH_2_) of LYS6 residue, respectively (Figs 2D and 3D). Additionally, it exhibited strong contacts with GLU13, LYS10, SER9 and LYS6 residues (Fig. 3D). These docking results reflected the potential binding of L-DOPA drug molecule and its interference with the function of with α-synuclein. Since, molecular interaction of CeO_2_ NP exhibited substantial contacts and interaction with α-synuclein at the active sites (Figs 2A and 3A), hence, CeO_2_ NP was deduced as potential inhibitor of α-synuclein.

### Replica exchange molecular dynamics simulation (REMD)

Replica-Exchange Molecular Dynamics (REMD) technique was employed to check the stable conformation of α-synuclein complexed with selected NPs and L-DOPA drug molecule through potential energies to sample conformations at two different temperatures (Fig. S1). Herein, two replicas were subjected to simulation at 300 K and 310 K for predicting the protein new stable conformational in space. Both replicas (300 K and 310 K) of CeO_2_ NP complex showed stable conformation in all the frames (200–1000 frames selected from 25 ns simulation trajectory) selected between 0.2–0.6 nm RMSD (Fig. S1A). Similar results were also recorded for the AuNP and SPION with different RMSD values between 0.6–1.5 nm and 0.6–1.4 nm, respectively (Fig. S1B,C). Also, L-DOPA confirms this replica exchange simulation which showed stable conformation between 0.6–1.2 nm (Fig. S1D). Large values of RMSD depicted the conformation changes in protein, however, the values between 0.6–1.3 nm for RMSD has been reported as stable range for α-synuclein.

**Figure 4:**
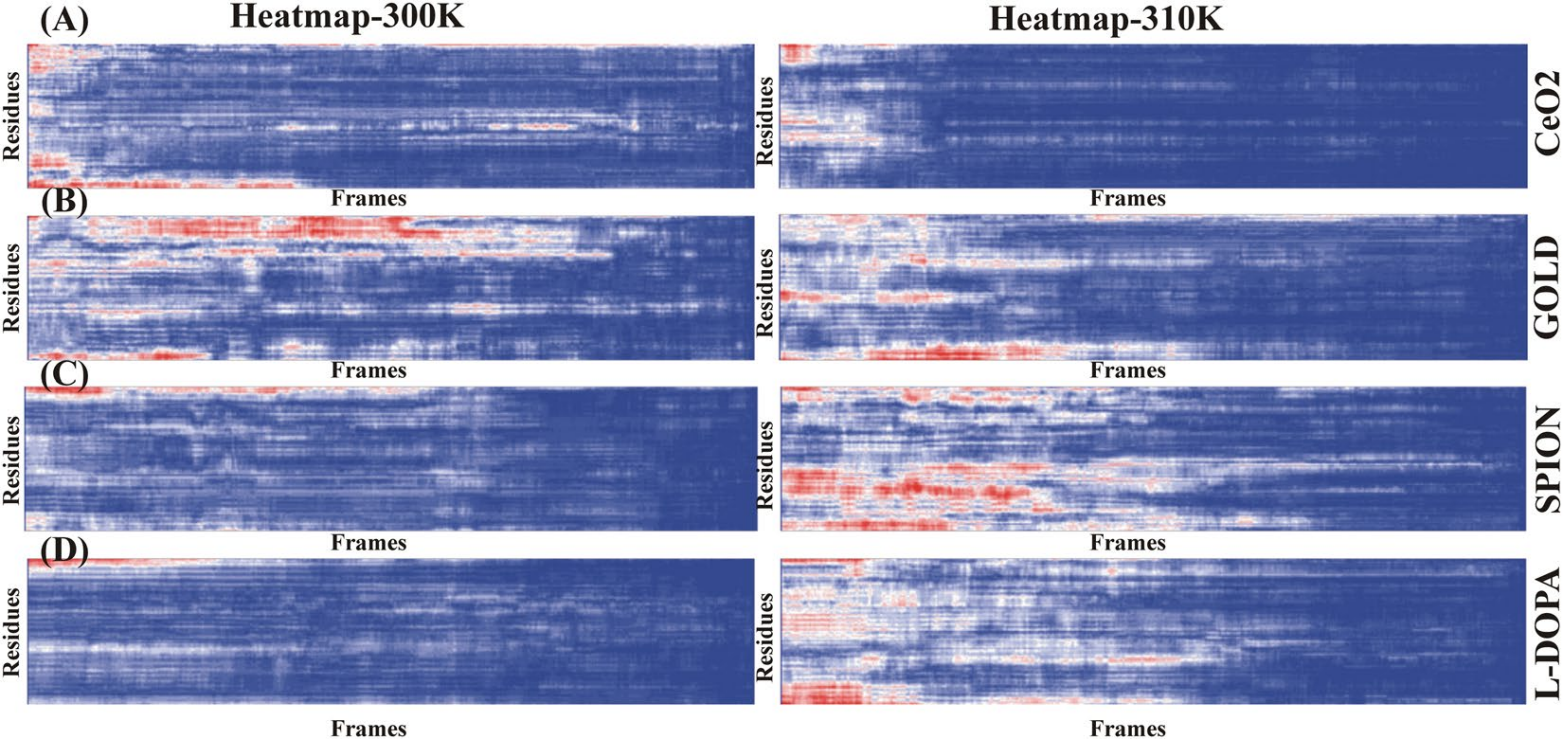
The heat map plot of two replica generated using REMD for 25 ns frames vs Residues. Blue color indicates each residue have stable state and stabilized the protein conformations during simulation in both replica and red indicate the fluctuations in residues level cause higher conformations during simulation (low to higher temperatures).

Also, the heat map was generated for REMD with the selected NPs and Standard drug L-DOPA complexed with α-synuclein at two different temperatures (Fig. 4). It was observed that CeO_2_ NP showed stable conformation in both the replicas as shown in blue color except some changes in starting frames (Fig. 4A). However, AuNP showed higher conformational changes in first replica while second replica it has stable state after 500 frames (Fig. 4B). Likewise, SPION was recorded with higher changes in conformation in second replica while first replica showed stable state similar to L-DOPA (Fig. 4D). These observations suggested that the CeO_2_ NP contained more stable state as compared to other NPs (Fig. 4A).

### Prediction of α-synuclein activity with CeO_2_ NPs and L-DOPA drug molecules

To predict the α-synuclein activity in presence of CeO_2_NP and L-DOPA drug molecule, respective MD simulation (200 ns simulation time for each complex) was designed and root mean square deviation (RMSD) and root mean square fluctuation (RMSF) was studied (Figs S2 and S3).

For MD simulation of α-synuclein protein with CeO_2_ NP, an aggregated complex was generated containing 13801 atoms with 4117 water atoms. Also, potential energy of simulated system was kept stable between −45700 to −45670.05 kcal/mol for 200 ns simulation time. The system volume, pressure and temperature were kept constant as 136399Å^3^, 1.190 bars and 298 K, respectively. RMSD analysis was applied to elucidate the protein backbone atoms flexibility and conformation changes during simulation with reference to initial structure. Also, we observed that binding conformation of α-synuclein protein with CeO_2_ NP showed stable and constant range of RMSD between 1.4–1.7 nm. Similarly, L-DOPA protein complex reflected RMSD values between 0.5–1.0 nm. These constant ranges of RMSD suggested that α-synuclein have stable state during long time simulation (200 ns).

### Biochemical pathway analysis of α-synuclein

PD biochemical pathway caused by α-synuclein was designed using SBGN (Systems Biology Graphical Notation) for representing the biological interactions, such as protein-protein interactions, signaling pathways and gene regulatory networks (Fig. S4) as well as by pharmacokinetics studies (Fig. S7). SBGN in PD biochemical pathway mainly focusses on three graphical notations: process diagram, entity relationship diagram and activity flow. It was followed by various components of biochemical pathway including species (genes, proteins, molecules, complex), reactions (forward, reverse) or compartments. For the expression of biochemical networks in PD, role of various interacting molecules or species have been demonstrated using various icons with different colors. Herein, green square shaped components labeled in various reactions represent the species such as proteins, simple molecules in the biochemical pathway of PD. The various inhibiting and transition states as well as reactions occurring between different connected species in biochemical pathway are represented by arrows. Moreover, these arrows also reflected the phenomenon of state transitions, known transition omitted, unknown transition, and transportation of various species. The correlation between various molecules of PD biochemical pathway and α-synuclein have been identified, annotated by some arrows and depicted the relationship between them. PD occurred in mammalian brain where dopamine acted as an important and functional neurotransmitter as it has been reported to control many functions such as locomotory activities, thinking skills, and other neurological activities. The biochemical pathway revealed that both CeO_2_ NP (Fig. S5) and L-DOPA drug molecule (Fig. S6) induced dopamine secretion from presynaptic axonal terminals and interact with five receptor subtypes in central nervous system included D1-like receptors (D1Rs) and D2-like receptors (D2Rs). D1Rs contained D1 and D5 receptors that acted positively for cAMP and adenylyl production while other D2 like receptors (D2Rs) consisted of D2, D3 and D4 receptors, their activation resulted into suppression of CAMP production and inhibition of adenylyl cyclase. Also, various calcium regulated processes like intracellular calcium levels and several calcium dependent intracellular signaling pathways were observed to controlled by D1 and D2 like receptors. Besides, dopamine also showed effects on neuronal activity, synaptic plasticity and behavioral environment through different CAMP, calcium dependent and independent mechanisms. Additionally, D2Rs located presynaptically maintained the synthesis and secretion of dopamine that acted as main auto receptor for the dopaminergic system. Interestingly, CeO_2_ NPs (Figs S5 and S8) showed significant inhibition of PD against L-DOPA drug (Figs S6 and S9) as observed from the respective complete biochemical pathways and supported by the pharmacokinetics, wherein significant reduction in entities participating for the cause of PD pathogenesis (Figs S4 and S7) was observed. In summary, following key points have been recorded and evaluated during the complete PD biochemical pathway analysis in presence of CeO_2_ NP nano-drug are as follow;

1. Activation of various dopamine receptors lead to suppression of CAMP production and hence, inhibition of adenylyl cyclase.
2. Also, calcium ions (Ca^2+^) acted as major component during the PD that further regulated the other pathways being activated by dopamine receptors. Also, It was further supported by the literature survey that documented the α-synuclein interaction with dopamine.
3. In this Parkinson’s biochemical pathway, PKA interacted with many other genes as well as α-synuclein directly interacted with it.

### Boolean Network analysis using Synthetic Biology

**Figure 5:**
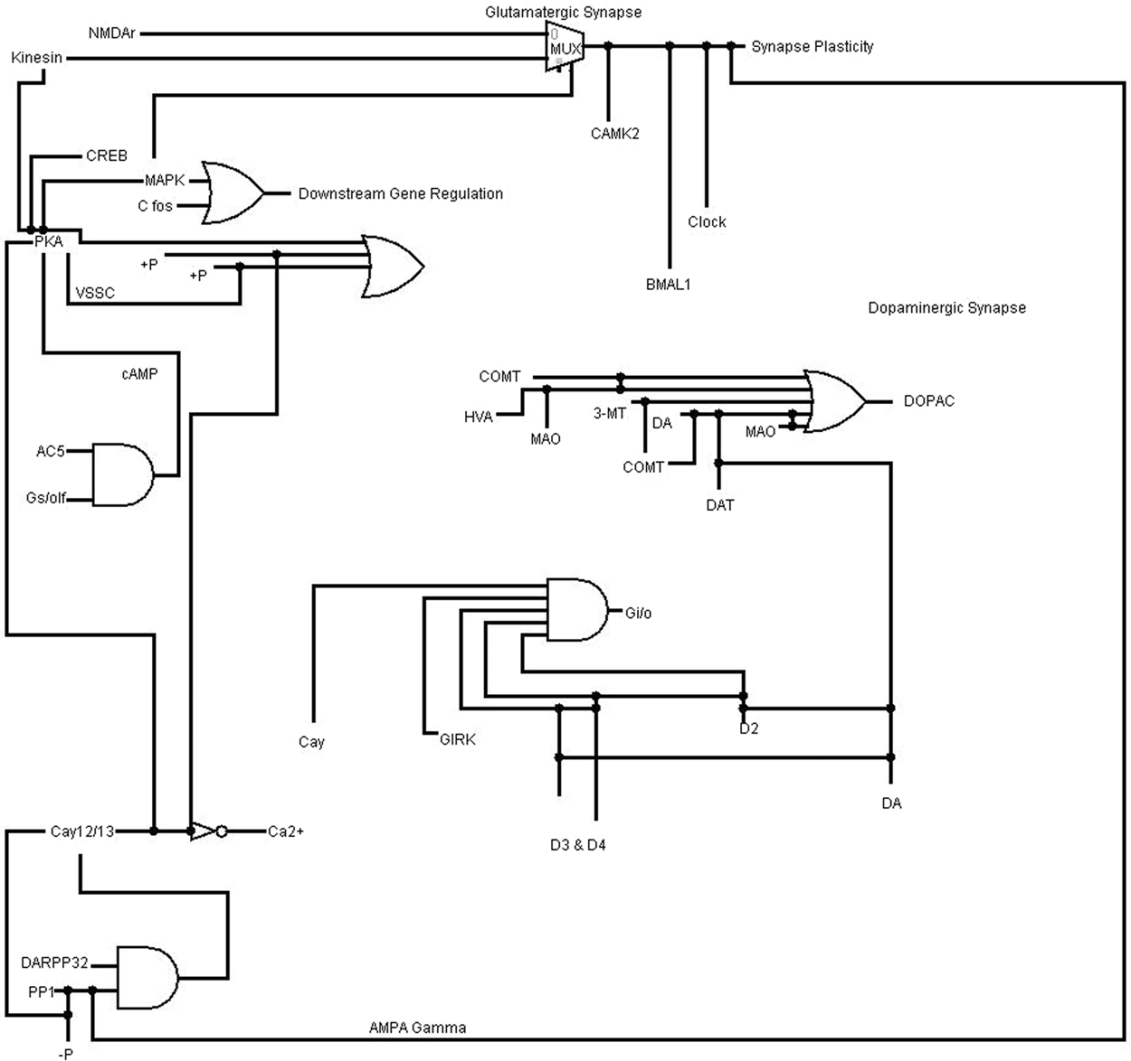
Biological circuit for PD mechanism and try to investigate the role of α-synuclein with CeO_2_ NP.

Using DNA computing approach, switching biology can be used to construct artificial Boolean network which can traverse whole regulatory mechanism. It’s based on biological circuit silicon chip implemented on A, C, T and G units that allows organisms to sense and control the regulatory systems. Biological circuit approach can analyse circuit behaviour and implement into developing RNA based post-transcriptional and translational control systems. Biological circuits classify and switch between cellular states as well as circuits to continually balance the states for efficient production. When the signal is “off”, the circuit is “off” and when signal is “on”, the circuit becomes responsive in a log-linear fashion. Here, we constructed the biological circuit for PD pathogenesis and tried to investigate the role of α-synuclein with CeO_2_ NP (Fig. 5). The construction of first feature for the α-synuclein with CeO_2_ NP was to inhibit the regulation of Parkinson mechanism in milliseconds to nanoseconds, even though turn of mechanism changes might be more rapidly. The black lines in Fig. 5 represent the 0 and 1 supply for every interacting molecules, whole circuits are combination of basic logic GATE.

## Methodology

### Molecular Modelling of Nanoparticles with α-synuclein

α-synuclein possess folded conformations and hence, it has been reported with many crystal structures in domain wise conformations which are available in protein databank as 2 X6 M, 3Q25, 3Q26, 3Q27, 3Q37, 3Q28, 3Q29, 4R0U, 4R0W, 4RIK and 5CRW. However, we retrieved the 3D structures of α-synuclein [PDB ID:1XQ8], a full length and stable NMR structure of Human micelle-bound α-synuclein conformation structure for our computational study, and minimized the structure using Amber99SB force field with default parameters. Based on biodegradability, biocompatibility and drug delivery across BBB as reported in literature, Cerium oxide (CeO_2_) (CID_73963), SPION (Fe_2_O_2_) (CID_16211978) and GOLD (Au) (CID_23985) NP were selected as nano-drug while L-DOPA (CID_6047) was selected as standard drug for docking with α-synuclein by Genetic algorithm from Autodock (using custom Atom type parameters for GOLD, SPION and CeO_2_) and compared with patch hotspot shape complementary algorithm approach to calculate the reliability of protein-ligand complexes^32,33^.

### Replica Exchange MD Simulation

To check the stable conformations of α-synuclein at different temperature conditions, each REMD simulations contained two replicas was performed for the docked complexes of α-synuclein with three NPs and L-DOPA by Desmondv4.4^34–36^. Since, α-synuclein exits at body average temperature between 300–310 K, and hence, REMD simulation were carried out at two different temperatures of 300 and 310 K with exchange time between the replicas was set to 25 ns. The initial conformations for REMD simulations were generated from the three NPs and L-DOPA drug complexes based on the “classical” MD simulations, and with other parameters under default mode. Further, stable conformations were analyzed based on the difference between RMSD of generated 700 trajectory frames on 25 ns interval for each complex.

### Molecular Dynamics simulation

Based on docking studies, molecular dynamics simulations were carried on structure of α-synuclein in presence of selected NPs as ligand and compared to L-DOPA drug as control with simulation time of 200 ns for each complex. Additionally, TIP4P water molecules were added to the system by applying minimization using 3000 steps of steepest descent algorithm followed by 5000 steps of conjugate gradient algorithm holding 120 kcal/mol threshold energy. The designed complexes had at least 6 Å × 6 Å × 6 Å buffer in each direction of the gradient box to permit the substantial fluctuations in the conformations during MD simulations. These simulations were conducted by employing Desmond v4.4 suite program^34–36^. Also, a constant pressure was applied using anisotropic diagonal position scaling on time step of 0.002 ps interval during MD simulations. Moreover, temperature of the system was subjected to gradual increment from 100 K to 300 K with 20 ps NPT reassemble at 1 atm target pressure. Besides, Berendsen algorithm^37^ was employed with a scaling factor time constant of 0.2 while Lennard-Jones cutoff value was set at 8 Å. Additionally, SHAKE^38^ constraints were also applied to all the chemical bonds involving hydrogen atoms. Nevertheless, all the simulations were executed under same conditions as that of equilibration procedure while density of system was maintained near 1 g/cm^3^, and OPLS v2005 force field was used in all the calculations.

### Systems Biology approach for Parkinson’s disease

Parkinson’s disease (PD) is one of the most frequent neurodegenerative disease occurred due to preferential and progressive degeneration in dopaminergic (DA) neurons of the (SN) portion of pars compacta located in the midbrain. Herein, designing and execution of PD signalling cascade was done from the earlier reported data. Briefly, literature survey was conducted to gather the information on α-synuclein with respect to biological interaction and all other entities participating in PD pathogenesis such as genes, truncating proteins, generic proteins, ions and other molecules. Following complete biochemical pathway was designed for the PD pathogenesis using Cell Designer software^39–41^, where nodes represent the entities and edges represent the connectivity of interacting molecules within the virtual cell. Furthermore, potential nano-drug, elucidated based on docking and MD simulation results, was also incorporated and tested against the L-DOPA drug molecules in the α-synuclein induced complete biochemical pathway of PD.

### Time Course Simulation analysis

Also, pharmacokinetic or time course simulation simulations were conducted in absence and presence of both the potential nano-drugs (0.50 µM) and standard L-DOPA drug molecules (0.50 µM) for the designed α-synuclein induced complete biochemical pathway of PD. The pharmacokinetic results were analysed with respect to transition time for the entire pathway of PD by employing irreversible simple Michaelis–Menten equation or mass action kinetics equation. These respective simulations assisted in predicting the biological interactions of entities involved in PD biochemical pathway.

### Boolean Network analysis using Synthetic Biology

Biological based Boolean circuit for the PD was designed by employing Logisim^42^. It depicts the interactions and simulation in terms of logic circuit for α-synuclein with potential nano-drug and other interacting entities involved in PD pathogenesis. This logic circuit was constructed based on the purposed complete biochemical pathway of PD pathogenesis, wherein input values were assigned in the form of 1 and 0 or True and False using basic input and output universal table.

## Electronic supplementary material

Supplementary Data 1

## References

[1] Sanchez-Mut JV (2016). Human DNA methylomes of neurodegenerative diseases show common epigenomic patterns. Translational psychiatry.

[2] Chen WW, Zhang X, Huang WJ (2016). Role of neuroinflammation in neurodegenerative diseases. Molecular medicine reports.

[3] Hirsch EC, Jenner P, Przedborski S (2013). Pathogenesis of Parkinson’s disease. Movement Disorders.

[4] De Lau L (2004). Incidence of parkinsonism and Parkinson disease in a general population The Rotterdam Study. Neurology.

[5] Lang AE, Lozano AM (1998). Parkinson’s disease. New England Journal of Medicine.

[6] Kalia K, V Kalia S (2010). L. & J McLean, P. Molecular chaperones as rational drug targets for Parkinson’s disease therapeutics. CNS & Neurological Disorders-Drug Targets (Formerly Current Drug Targets-CNS & Neurological Disorders).

[7] Trojanowski, J. Q., Goedert, M., Iwatsubo, T. & Lee, V. M. Fatal attractions: abnormal protein aggregation and neuron death in Parkinson’s disease and Lewy body dementia. *Cell Death & Differentiation* **5** (1998). 10.1038/sj.cdd.4400432 10203692

[8] Simon-Sanchez J (2009). Genome-wide association study reveals genetic risk underlying Parkinson’s disease. Nature genetics.

[9] Satake W (2009). Genome-wide association study identifies common variants at four loci as genetic risk factors for Parkinson’s disease. Nature genetics.

[10] Ren Z (2015). Neuroprotective effects of 5-(4-hydroxy-3-dimethoxybenzylidene)-thiazolidinone in MPTP induced Parkinsonism model in mice. Neuropharmacology.

[11] Cranwell-Bruce LA (2010). Drugs for Parkinson’s disease. MedSurg Nursing.

[12] Connolly BS, Lang AE (2014). Pharmacological treatment of Parkinson disease: a review. Jama.

[13] Hung AY, Schwarzschild MA (2007). Clinical trials for neuroprotection in Parkinson’s disease: overcoming angst and futility?. Current opinion in neurology.

[14] Volkman, R. & Offen, D. Concise Review: Mesenchymal Stem Cells in Neurodegenerative Diseases. *Stem Cells* (2017). 10.1002/stem.2651 28589621

[15] Rice J, Antic R, Thompson PD (2002). Disordered respiration as a levodopa‐induced dyskinesia in Parkinson’s disease. Movement disorders.

[16] Jenner P (2008). Molecular mechanisms of L-DOPA-induced dyskinesia. Nature reviews. Neuroscience.

[17] Spuch C, Saida O, Navarro C (2012). Advances in the treatment of neurodegenerative disorders employing nanoparticles. Recent patents on drug delivery & formulation.

[18] Schlachetzki F, Zhang Y, Boado RJ, Pardridge WM (2004). Gene therapy of the brain The trans-vascular approach. Neurology.

[19] Pardridge WM (2006). Molecular Trojan horses for blood–brain barrier drug delivery. Current opinion in pharmacology.

[20] Álvarez YD (2013). Influence of gold nanoparticles on the kinetics of α-synuclein aggregation. Nano letters.

[21] Mahmoudi M, Akhavan O, Ghavami M, Rezaee F, Ghiasi SMA (2012). Graphene oxide strongly inhibits amyloid beta fibrillation. Nanoscale.

[22] Padmanabhan P, Kumar A, Kumar S, Chaudhary RK, Gulyás B (2016). Nanoparticles in practice for molecular-imaging applications: an overview. Acta biomaterialia.

[23] Mirsadeghi S (2015). Protein corona composition of gold nanoparticles/nanorods affects amyloid beta fibrillation process. Nanoscale.

[24] Hellstrand E, Boland B, Walsh DM, Linse S (2009). Amyloid β-protein aggregation produces highly reproducible kinetic data and occurs by a two-phase process. ACS chemical neuroscience.

[25] Mahmoudi M (2010). Magnetic resonance imaging tracking of stem cells *in vivo* using iron oxide nanoparticles as a tool for the advancement of clinical regenerative medicine. Chemical Reviews.

[26] Hegazy, M. A. E. *et al*. The possible role of cerium oxide (CeO 2) nanoparticles in prevention of neurobehavioral and neurochemical changes in 6-hydroxydopamine-induced parkinsonian disease. *Alexandria Journal of Medicine* (2017).

[27] Schubert D, Dargusch R, Raitano J, Chan S-W (2006). Cerium and yttrium oxide nanoparticles are neuroprotective. Biochemical and biophysical research communications.

[28] Kaushik AC, Sahi S (2015). Boolean network model for GPR142 against Type 2 diabetes and relative dynamic change ratio analysis using systems and biological circuits approach. Systems and synthetic biology.

[29] Zhang B, Tian Y, Zhang Z (2014). Network biology in medicine and beyond. Circulation: Cardiovascular Genetics.

[30] Wu X, Al Hasan M, Chen JY (2014). Pathway and network analysis in proteomics. Journal of theoretical biology.

[31] Anderson JC, Clarke EJ, Arkin AP, Voigt CA (2006). Environmentally controlled invasion of cancer cells by engineered bacteria. Journal of molecular biology.

[32] Duhovny, D., Nussinov, R. & Wolfson, H. J. in *International Workshop on Algorithms in Bioinformatics*. 185-200 (Springer).

[33] Schneidman-Duhovny D, Inbar Y, Nussinov R, Wolfson HJ (2005). PatchDock and SymmDock: servers for rigid and symmetric docking. Nucleic acids research.

[34] Shivakumar D (2010). Prediction of absolute solvation free energies using molecular dynamics free energy perturbation and the OPLS force field. Journal of chemical theory and computation.

[35] Guo Z (2010). Probing the α‐helical structural stability of stapled p53 peptides: molecular dynamics simulations and analysis. Chemical biology & drug design.

[36] Bowers, K. J. *et al*. in *Proceedings of the2006 ACM/IEEE conference on Supercomputing*. 84 (ACM).

[37] Kräutler V, Van Gunsteren WF, Hünenberger PH (2001). A fast SHAKE algorithm to solve distance constraint equations for small molecules in molecular dynamics simulations. Journal of computational chemistry.

[38] Jorgensen WL, Tirado-Rives J (1988). The OPLS [optimized potentials for liquid simulations] potential functions for proteins, energy minimizations for crystals of cyclic peptides and crambin. Journal of the American Chemical Society.

[39] Funahashi A, Morohashi M, Kitano H, Tanimura N (2003). CellDesigner: a process diagram editor for gene-regulatory and biochemical networks. Biosilico.

[40] Funahashi A, Jouraku A, Matsuoka Y, Kitano H (2007). Integration of CellDesigner and SABIO-RK. In silico biology.

[41] Funahashi A (2008). CellDesigner 3.5: a versatile modeling tool for biochemical networks. Proceedings of the IEEE.

[42] Burch C (2002). Logisim: a graphical system for logic circuit design and simulation. Journal on Educational Resources in Computing (JERIC).

