# Supplementary material for: Nano-particle mediated inhibition of Parkinson’s disease using computational biology approach
Source: Sci Rep. 2018 Jun 15;8:9169. doi: 10.1038/s41598-018-27580-1 (PMC6003935; doi:10.1038/s41598-018-27580-1)
Supplement: Supplementary file 1 — Supplementary Data 1 [file 41598_2018_27580_MOESM1_ESM.docx]

**Nano-particle mediated inhibition of** **Parkinson's disease using computational biology approach**

Aman Chandra Kaushik^1^, Shiv Bharadwaj^2^, Sanjay Kumar^3^ and Dong-Qing Wei^*,1^

^1^State Key Laboratory of Microbial Metabolism and School of life Sciences and Biotechnology, Shanghai Jiao Tong University, Shanghai 200240, China

^2^Sabanci University Nanotechnology Research and Application Center, Orta Mah. Tuzla 34956, Istanbul, Turkey

^3^Bioinformatics Centre, Biotech Park, Lucknow 226018, India

***Corresponding authors**

DQW; Email: dqwei@sjtu.edu.cn; Phone +86-34204573

**S1. Results and discussion**

**S1.1. Molecular interaction of nanoparticles with α-synuclein**

The shape complimentary algorithm works similar to shape based virtual screening of number of compounds using active pocket area of protein and has been reported with potent results^1-4^. We analyzed the docked position of NPs at the active sites of α-synuclein using Autodock and compared the results against the conformations obtained by hotspot shape complimentary method from PatchDock server. This lead to selection of common top 20 conformations for each docked complex of nano-drug i.e. NPs and drug molecule generated from Autodock and PatchDock server. The calculated binding energy of CeO_2_ NP, L-DOPA and SPION showed acceptable range of docking energy while AuNP was recorded with least suitable binding energy, suggested due to lack of any type of interactions with α-synuclein. Similar results were also recorded for the NPs and drug molecule from the patch dock Atomic contact energy (Table S1). These comparative results validated the docked position for all the docked NPs and drug molecule stable binding conformations with protein. The final conformations were then selected from the AutoDock results and further subjected to molecular dynamics simulation studies.

**Table S1.** Docking results of α-synuclein protein with CeO_2_ NP, Au NP, SPION and L-DOPA drug molecules

| **Ligands** | **Active area** | **Binding energy** | | **Interacting residues** | |
| --- | --- | --- | --- | --- | --- |
|  |  | **∆G** | **ACE** | **Autodock** | **Patch dock** |
| CeO_2_ NP | 113.80 | -2.1 | 4.85 | GLY7, LEU8, PHE4 | VAL3, MET5, GLY7, LEU8, PHE4 |
| L-DOPA  Drug | 282.80 | -3.5 | -35.01 | SER9, MET5, GLY7, LYS10, GLU13, LYS12 | GLY7, LEU8, MET5, LYS6, GLU13, GLY14, VAL16 |
| *AuNP | 40.70 | -0.4 | 0.0 | PHE4, MET5, GLY7, LEU8 | PHE4, MET5, GLY7, LEU8, ALA11, LEU12 |
| *SPION | 135.50 | -2.8 | 62.94 | ALA11, LEU8, SER9, LYS12, LYS6 | VAL16, LYS12, GLU13, LYS10, LYS6, GLY7 |

** NPs taken as reference/control nanoparticles*

Note: *∆G-Lowest binding energy and ACE – Atomic Contact Energy*

**S1.2. Replica exchange molecular dynamics simulation (REMD).**

| 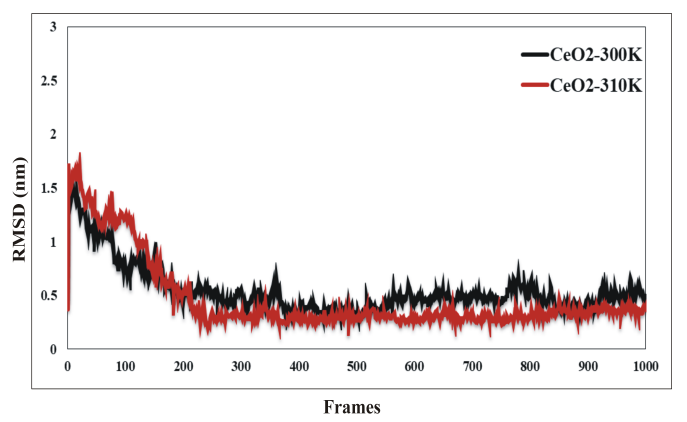  **(A)** | 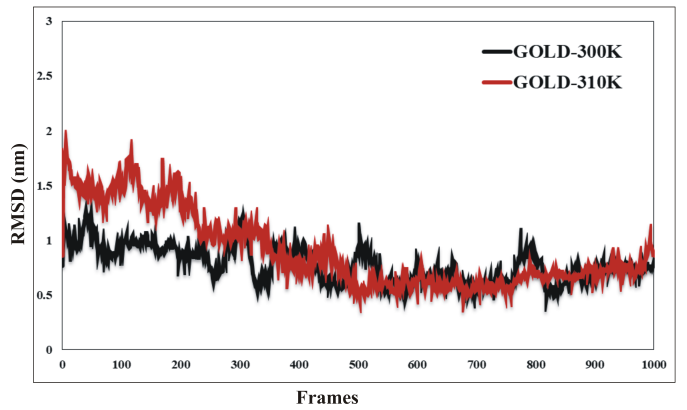  **(B)** |
| --- | --- |
| 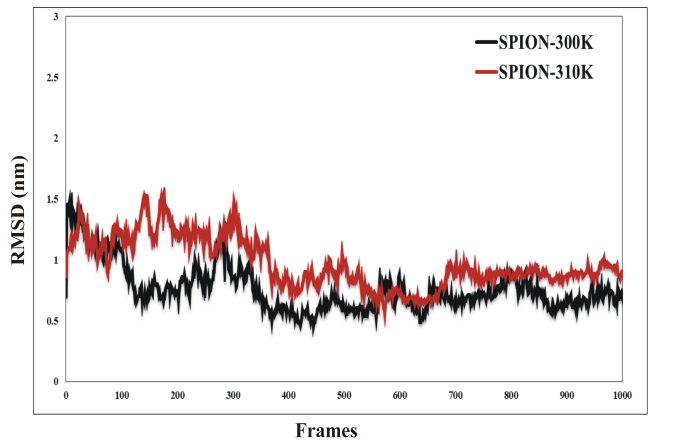  **(C)** | 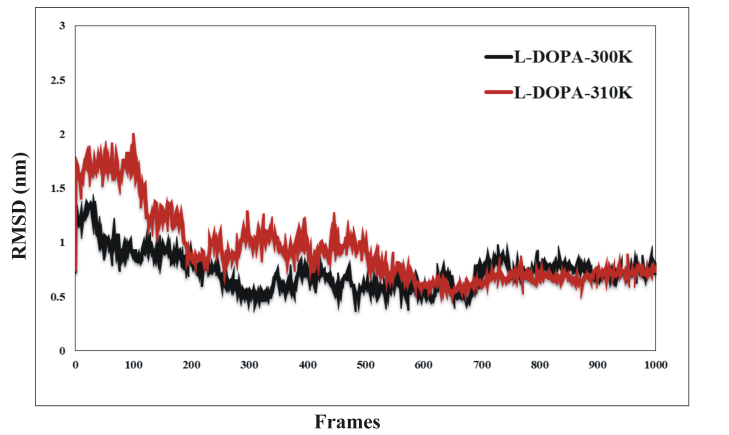  **(D)** |

**Figure S1.** RMSD difference between two replicas of NPs (A) CeO2, (B) AuNPs, (C) SPION and (D) L-DOPA drug. Two replicas (300 and 310K) were analyzes based on RMSD of each REMD complexes backbone atoms vs generated frames during simulation.

**S1.3.** **Prediction of α-synuclein activity with CeO_2_ NPs and L-DOPA drug molecules**


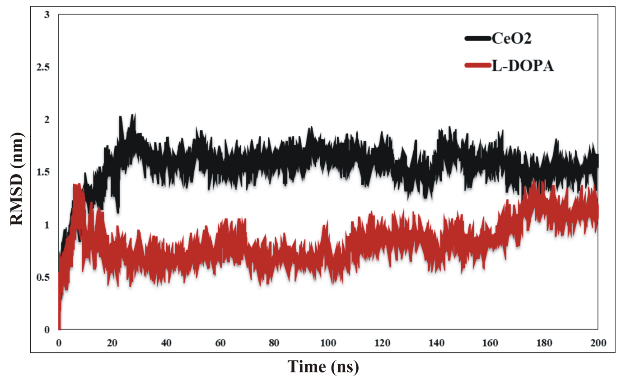


**Figure S2.** Protein backbone RMSD of α-synuclein with CeO_2_ NP and L-DOPA complex.

Furthermore, RMSF fluctuations were monitored for each residue to determine CeO_2_ NP and L-DOPA drug molecule impact on α-synuclein protein dynamic force behavior (Fig. S3). The results showed that the fluctuations in residue level was quite high as 38-43, 68-73 and 95-100, respectively for the CeO_2_ NP complex up to 8Å (Fig.S3a) while with L-DOPA drug molecule complex, these regions showed much higher values of 9Å to 10Å (Fig. S3b) against CeO_2_ NP complex. The greatest flexibility was recorded in the interacted region of CeO_2_ NP binding below 8Å to 4Å (Fig. S3a), while in L-DOPA drug molecule complex was measured with the flexibility between 12Å to 5Å (Fig. S3b).


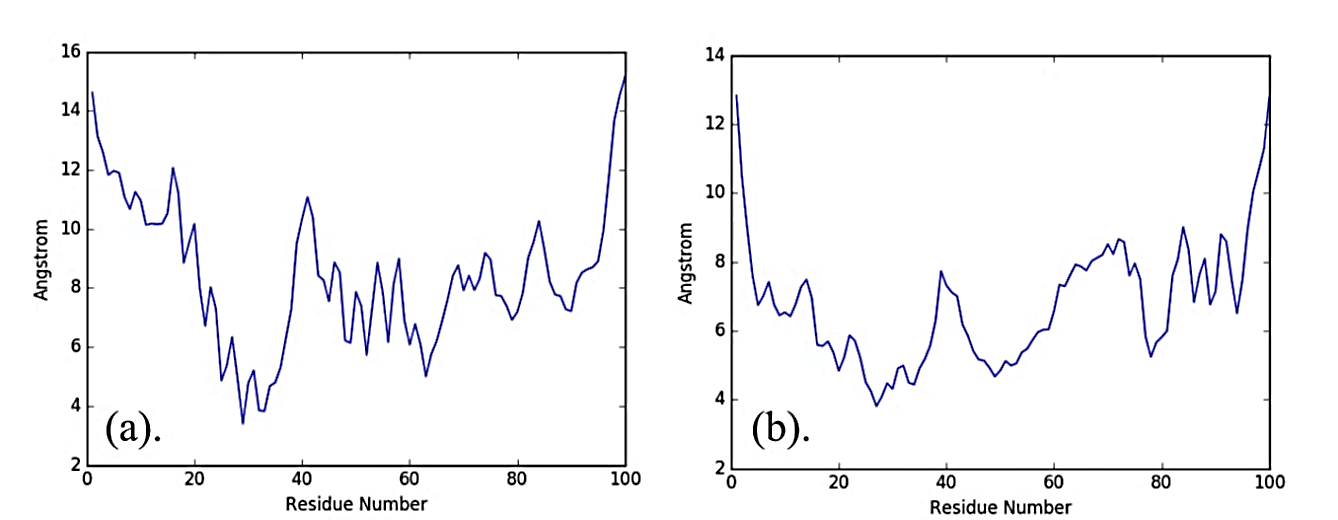


**Figure S3.** RMSF plot for (a) CeO_2_ NP and (b) L-DOPA complex during simulation.

**S1.4. Biochemical pathway analysis of α-synuclein**


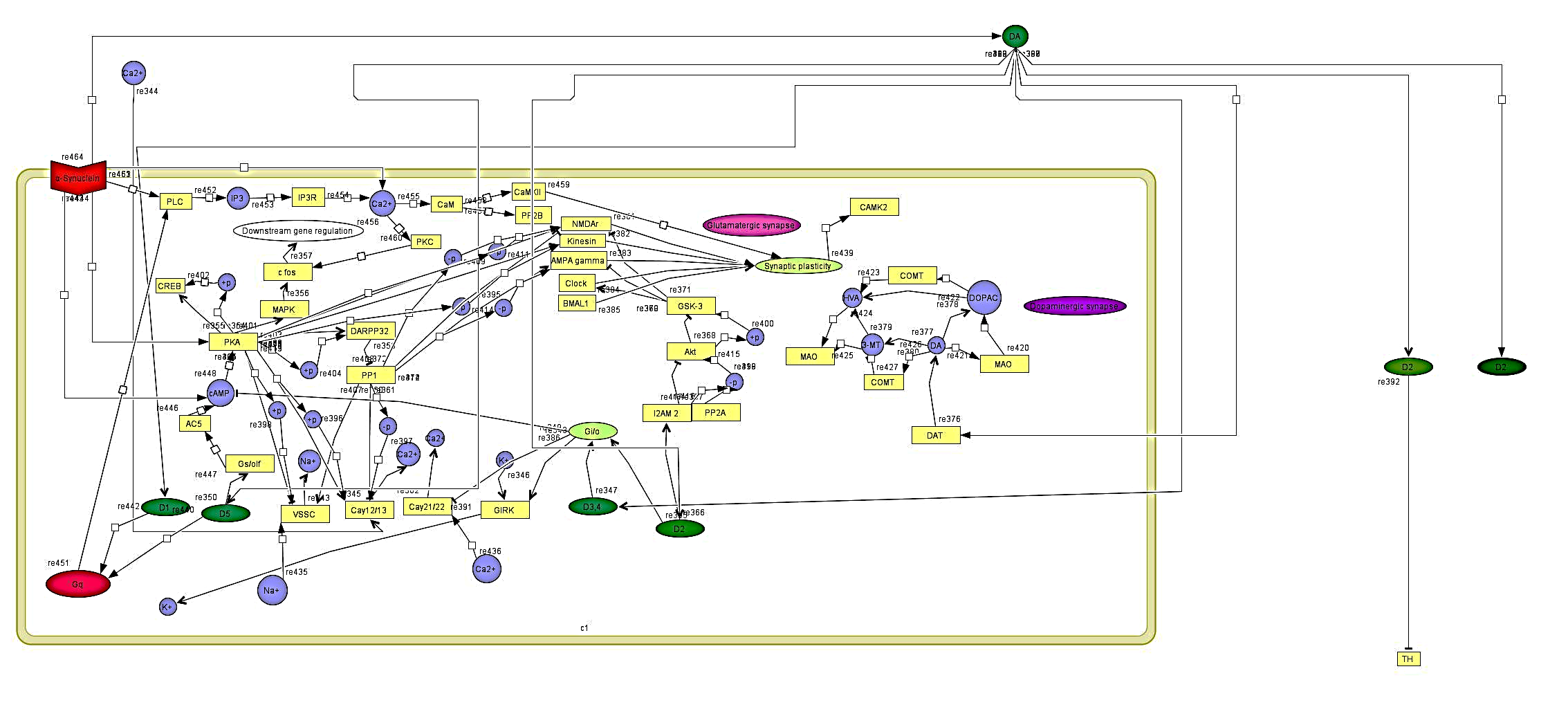


**Figure S4**. Biochemical pathway analysis in presence of α-synuclein without any nanoparticles indicates the dynamic changes after time course simulation.


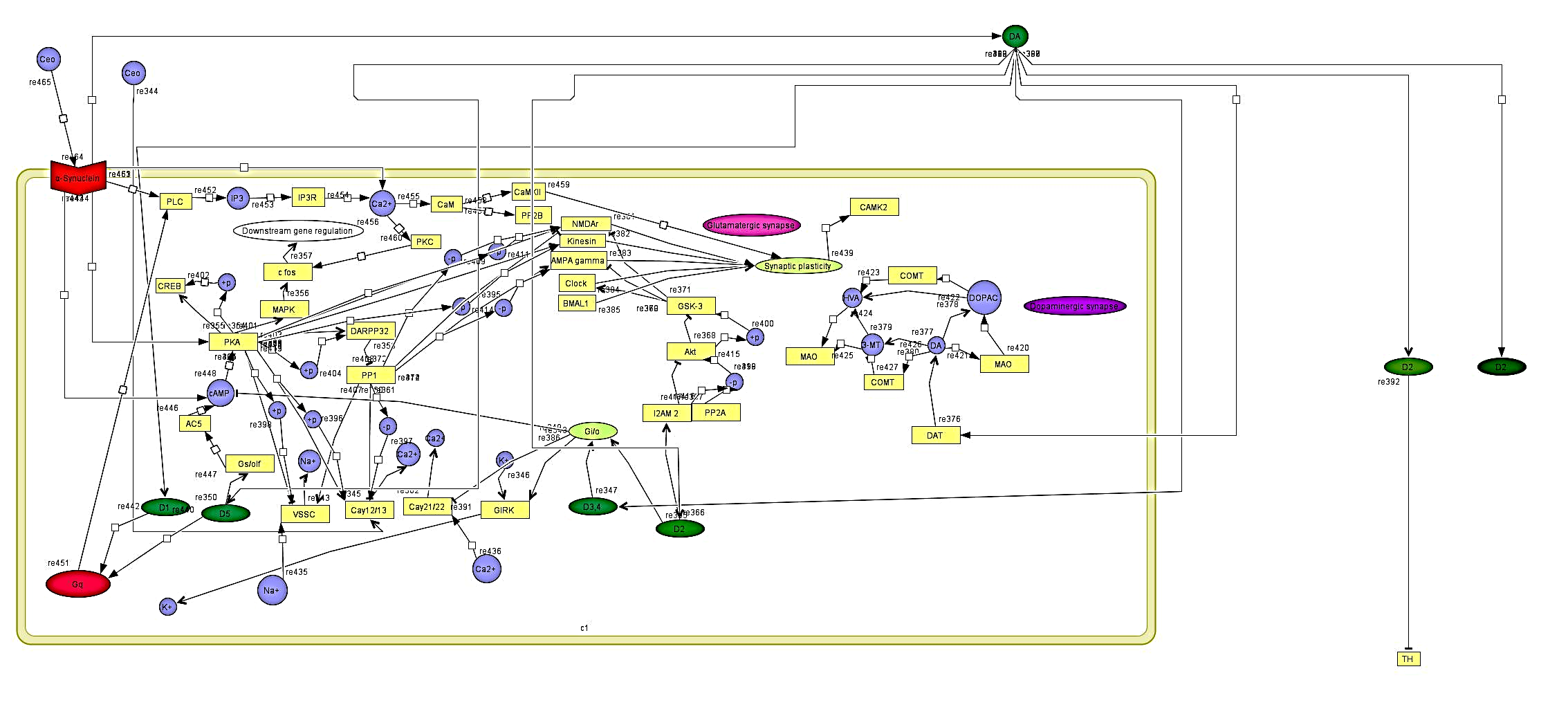


**Figure S5.** Biochemical pathway analysis in presence of CeO_2_ nanoparticles interacted with α-synuclein indicated the dynamic changes in the PD pathogenesis after time course simulation.


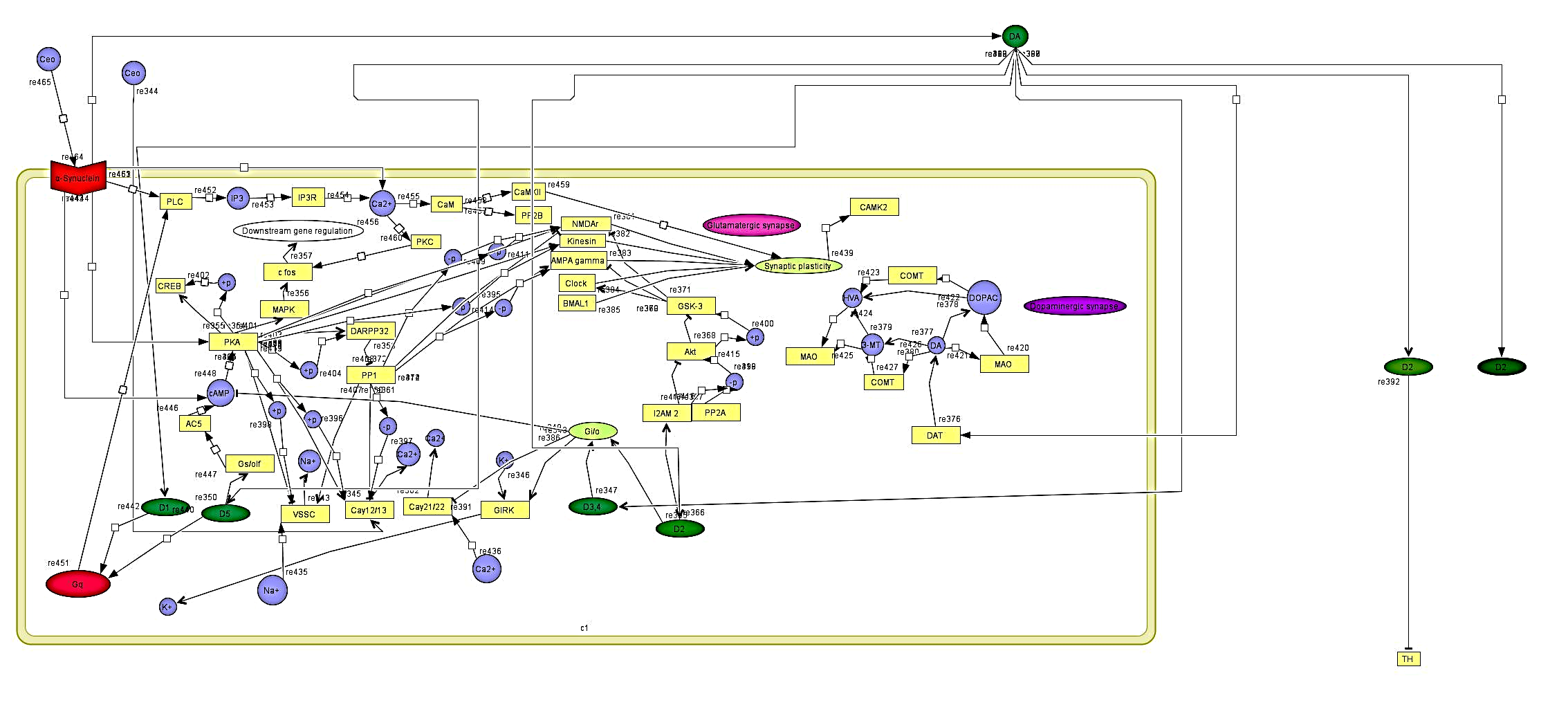


**Figure S6**. Biochemical pathway analysis in presence of L-DOPA drug molecules interacted with α-synuclein indicated the dynamic changes in the PD pathogenesis after time course simulation.

.


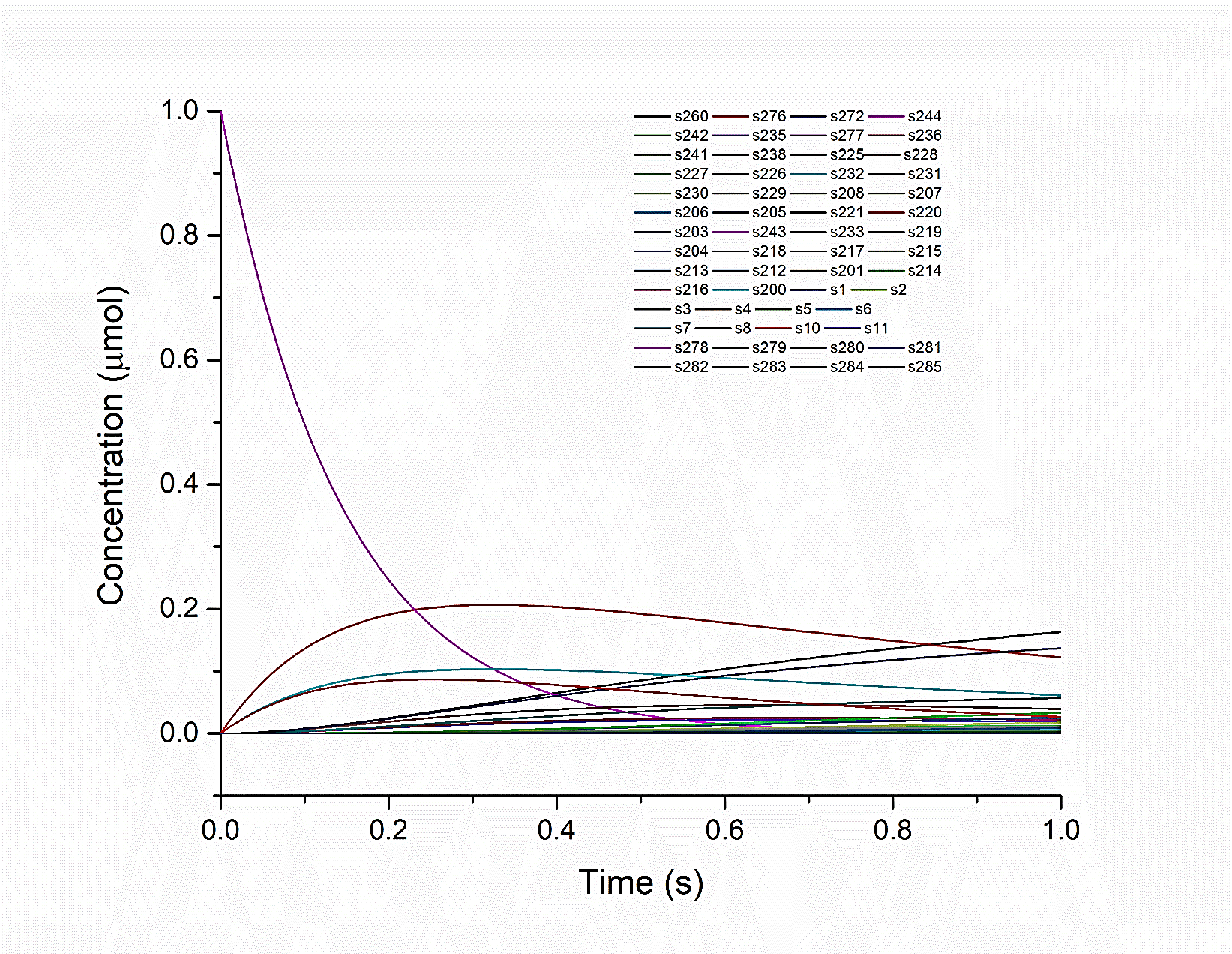


**Figure S7**. Time course simulation analysis of biochemical pathway in presence of α-synuclein where different colored lines are indicating the amount of different species, where X axis represents the time and Y axis represents the amount (concentration) of species. Entities (species are shown in different colors) and s1, s2... indicated the relationship between one species to other.


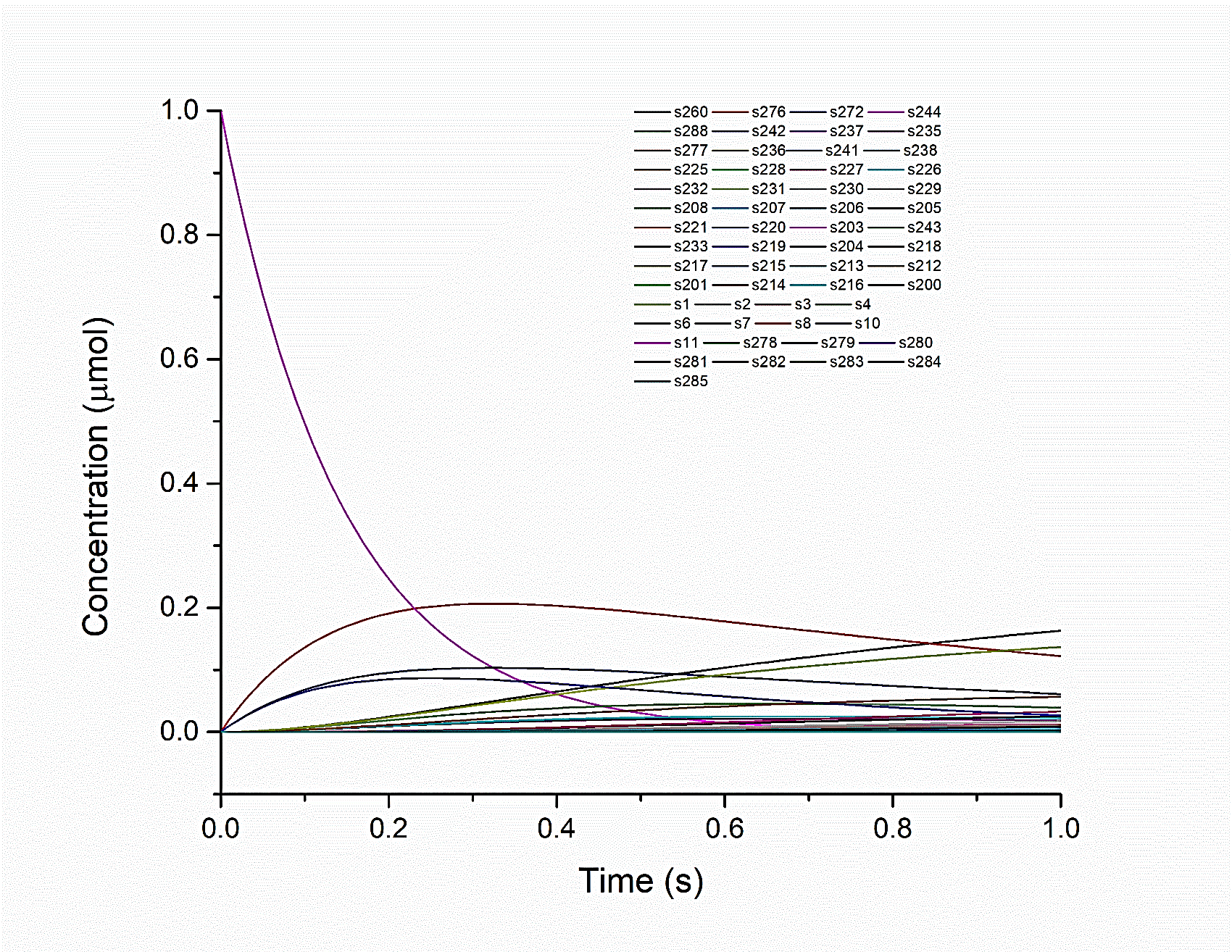


**Figure S8**. Time course simulation analysis in presence of α-synuclein with CeO_2_ NP where different colored lines are indicating the amount of different species, where X axis represents the Time and Y axis represents the amount (concentration) of species. Entities (species are shown in different colors) and s1, s2... indicated the relationship between one species to other.


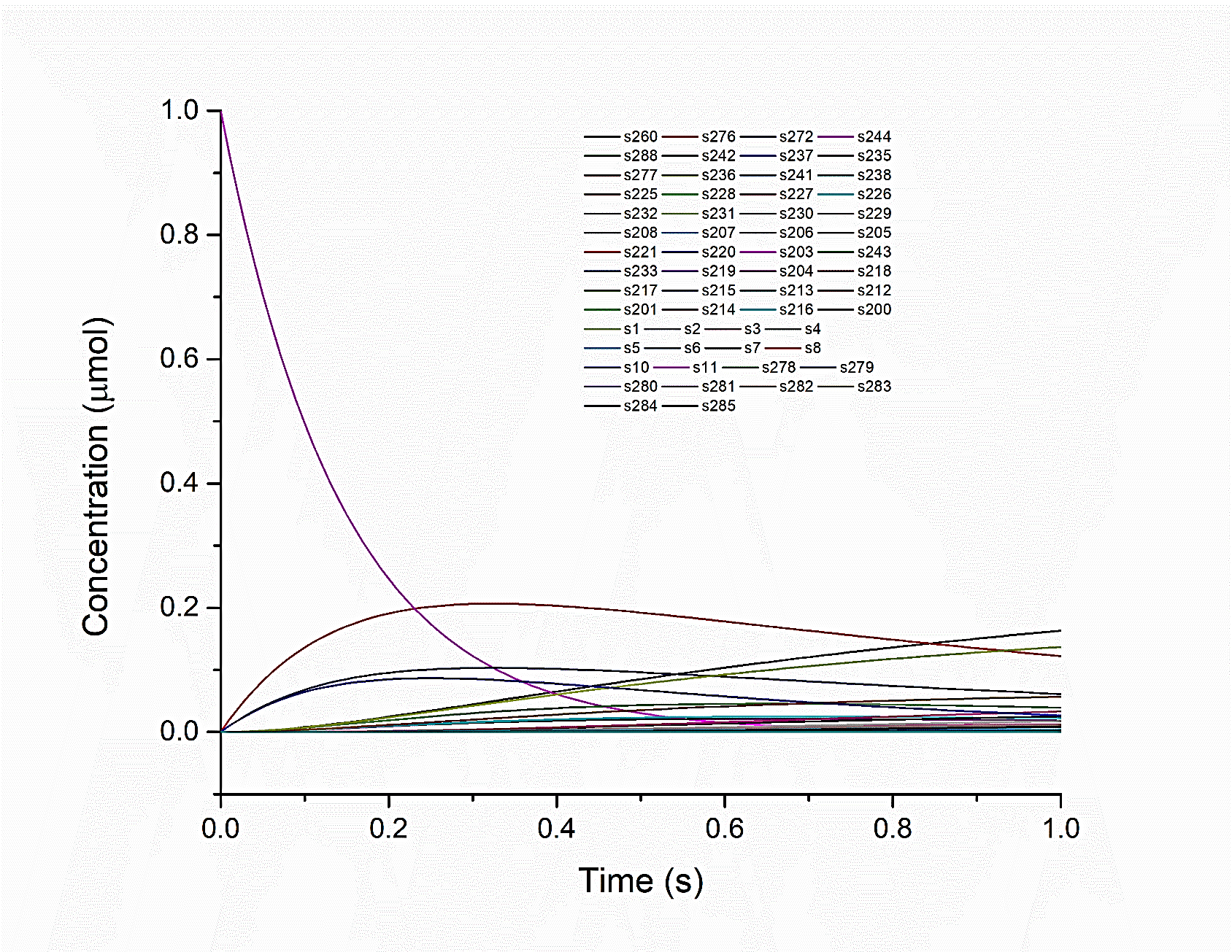


**Figure S9.** Time course simulation analysis in presence of α-synuclein with L-DOPAdrug molecule where different colored lines are indicating the amount of different species, where X axis represents the Time and Y axis represents the amount (concentration) of species. Entities (species are shown in different colors) and s1, s2... indicated the relationship between one species to other.

**References**

1 Autiero, I., Costantini, S. & Colonna, G. Human sirt-1: molecular modeling and structure-function relationships of an unordered protein. *PloS one* **4**, e7350 (2009).

2 Kumar, U. C. *et al.* Discovery of novel InhA reductase inhibitors: application of pharmacophore-and shape-based screening approach. *Future medicinal chemistry* **5**, 249-259 (2013).

3 Maganti, L., Consortium, O. & Ghoshal, N. 3D-QSAR studies and shape based virtual screening for identification of novel hits to inhibit MbtA in Mycobacterium tuberculosis. *Journal of Biomolecular Structure and Dynamics* **33**, 344-364 (2015).

4 Temml, V., Voss, C. V., Dirsch, V. M. & Schuster, D. Discovery of new liver X receptor agonists by pharmacophore modeling and shape-based virtual screening. *Journal of chemical information and modeling* **54**, 367-371 (2014).
